# A student-led interprofessional virtual outreach program for people with HIV during the Covid-19 pandemic: a pilot program at an academic medical center in Boston

**DOI:** 10.1186/s12909-022-03716-w

**Published:** 2022-09-02

**Authors:** Daniel A. Solomon, Susan Larrabee, Joshua Ellis, Parsa Erfani, Shawn F. Johnson, Katherine M. Rich, Raquel Sofia Sandoval, Nora Y. Osman

**Affiliations:** 1 Division of Infectious Diseases, Brigham and Women’s Hospital, 75 Francis Street PBB-4A, Boston, MA 02115 USA; 2 Harvard Medical School, Boston, MA USA; 3 Department of Medicine, Brigham and Women’s Hospital, 75 Francis Street PBB-4A, Boston, MA 02115 USA

**Keywords:** Clinical skills, Communication skills, Undergraduate, Interprofessional

## Abstract

**Background:**

The Coronavirus disease 2019 (Covid-19) pandemic caused an abrupt disruption in clinical care and medical education, putting patients at increased risk for social stressors and displacing medical students from traditional clerkships. The pandemic also exposed the need for virtual tools to supplement clinical care and an opportunity to create meaningful roles for learners.

**Methods:**

An interdisciplinary group designed a student-led virtual outreach program for patients with HIV whose care was limited by the pandemic. Patients were identified by clinicians and social workers using a clinic-based registry. Students called patients to conduct needs assessments, provide Covid-19 education, and to facilitate connection to services. Students participated in case-based didactics and workshops on motivational interviewing and patient engagement using virtual tools. Facilitated team meetings were held weekly during which themes of calls were identified.

**Results:**

During a three-month period, five students participated in the outreach program. Two hundred sixteen patients were identified for outreach calls, of which 174 (75.9%) were successfully reached by telephone. Rate of completed phone call did not differ by age or gender. Sixty patients had a preferred language other than English of which 95.6% were reached in their preferred language.

**Conclusions:**

Virtual proactive outreach can be used as a tool to support patients and engage students in clinical care when access to in-person care is limited. This model of care could be adapted to other ambulatory practices and integrated into pre-clerkship curriculum as an introduction to the social history and structural drivers of health (SDOH) (245/350).

**Supplementary Information:**

The online version contains supplementary material available at 10.1186/s12909-022-03716-w.

## Background

The Coronavirus disease 2019 (Covid-19) pandemic caused an abrupt disruption in both health care delivery and medical education. Healthcare centers and medical schools closed both clinical spaces and classrooms, limiting access for patients and students. Physicians and hospitals developed models of virtual care and telehealth to fill gaps in clinical care [1, 2]. In parallel, medical schools modified curricula and teaching methods to engage learners on virtual platforms [3, 4]. Despite these interventions, patients living with chronic diseases saw reductions in their access to non-urgent care, prevention and support.

We describe the pilot of a student-led virtual outreach program that engaged students in direct patient care during a time when they were displaced from traditional clerkships. The pilot was designed to identify and address structural drivers of health (SDOH) in an outpatient Human Immunodeficiency Virus (HIV) clinic during the early stages of the Covid-19 pandemic. People with HIV are at increased risk for social isolation due to HIV-related stigma, both experienced and perceived [5, 6]. Stigma can lead individuals with HIV to withdraw from social relationships and networks as a means of concealing HIV status and avoiding disclosure [7]. Physical distancing measures in response to Covid-19 may further increase risk of social isolation in this population [8].

The outreach program was designed to identify needs in this socially vulnerable population and engage students in direct patient care, creating a value-added [9] role for students on the care team. The pilot also included interactive clinical skills workshops on the principles of motivational interviewing and patient engagement using virtual tools, as well as case-based didactics on Covid-19.

## Methods

### Program development

In April 2020, five second-year medical students who were displaced from their respective traditional clerkships due to Covid-19 restrictions volunteered to participate in a health outreach program for medically and socially vulnerable patients.

Although their first-year curriculum included content on social determinants of health, the students previously had no formal training on how to engage patients in conversations about structural drivers of health and limited practical knowledge regarding local social resources. The students had limited to no experience in telehealth. One student had experience working with people with HIV at a needle exchange program, but otherwise they did not have experience in caring for and counseling patients with HIV.

In collaboration with a social worker (S.L.) and an infectious disease physician (D.A.S.) the students designed a telephone outreach program for patients with HIV connected to care at the Infectious Diseases Clinic at Brigham and Women’s Hospital (BWH) with the following goals:

1. Screen vulnerable patients for acute medical and social needs during the pandemic.
2. Provide patients individualized Covid-19 education.
3. Provide direct referral to social services, including food assistance and low-barrier, easy-access Covid-19 testing.
4. Provide students an opportunity for direct patient care within an interprofessional framework.
5. Teach students principles of motivational interviewing with opportunity for practice, observation, and feedback.
6. Offer students real-time Covid-19 clinical updates in the context of the rapidly evolving pandemic.

To standardize outreach calls and provide language and screening tools around structural drivers of health (SDOH), the physician and social worker developed a semi-structured script based on principles of motivational interviewing (Additional file 1) [10]. Three students were monolingual English speakers, two students were bilingual in English and Spanish, and one student was bilingual in English and Haitian Creole. This allowed for English, Spanish, and Haitian Creole-speaking patients to receive language-concordant outreach. For discordant language pairings, students used a licensed medical interpreter to communicate with patients.

### Process (Fig. 1)

The BWH infectious disease clinic cares for 1080 patients with HIV. 37% self-identify as Black or African American and 13.1% identify as Hispanic. Using a clinic registry, clinicians and social workers identified patients as candidates for outreach calls based on prior history of need for social support or history of social isolation with a goal of providing outreach to 20% of the clinic population (216 patients).

**Fig. 1:**
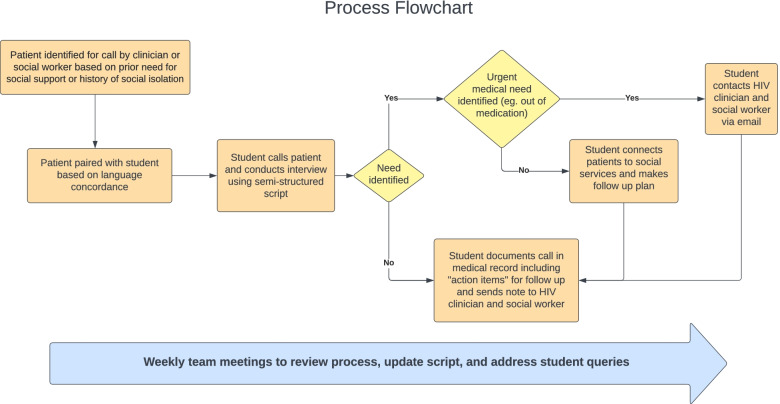
Process Flowchart. Legend: Orange boxes represent steps in the workflow; yellow triangles represent key decision points

The students each called five unique patients every week. The students made three attempts to contact each patient. Voicemails explaining the program were left when possible. Students made initial connections to food assistance, social services, and Covid-19 testing during calls. Patients expressing a desire for more social connection were offered information about virtual support groups. The student entered a note summarizing the call with “action items” for follow up into the electronic medical record and sent the note to the patient’s social worker and physician. We sent face masks to all patients who were unable to obtain one independently. Members of the team met virtually every week to debrief the outreach calls, troubleshoot challenges, revise the semi-structured script based on evolving patient needs, and evaluate student experiences.

The weekly team meetings began with a didactic session tailored to the needs of the program and the interests of the students. The educational focus at the beginning of the pilot was the development of clinical skills through interactive sessions on the principles of motivational interviewing and best practices for engaging patients around structural drivers of health in the virtual setting. The focus of the sessions then shifted to clinical updates on Covid-19 including epidemiology, clinical manifestations of infection, transmission dynamics and disease prevention, impact of Covid-19 on patients with HIV, novel therapeutics, and the evolving landscape of diagnostic testing.

### Program evaluation

We reviewed all notes from student outreach calls conducted between April 1, 2020 and July 15, 2020 to evaluate the scope of outreach to patients. We recorded data on patients’ age, gender, and preferred health care language. We defined a successful outreach attempt to patients as confirmed contact with the patient. In order to analyze the completion of calls across the age spectrum, patients were divided into two age groups, age < 60 or age ≥ 60 to determine whether successful outreach attempt differed between younger and older individuals. The BWH IRB reviewed the project and deemed it exempt.

Students took personal notes during their calls and documented formal notes in the electronic health record (EHR). During weekly meetings with faculty leads students discussed and identified recurring themes that emerged during outreach calls based on their recall, personal notes, and the notes documented in the EHR. The themes were agreed upon by all students. These themes included the impact of the program on patients, students, and clinicians and are described further in the results section below. Some of the themes emerged directly from outcomes such as connection to food services, others were based on direct comments from patients during phone calls.

## Results

Students called a total of 216 patients (20% of the clinic population) and successfully reached 75.9%. Rate of successful outreach did not significantly differ by gender. Of 100 patients who self-identified as female, 77 (77%) were reached successfully; of 115 patients who self-identified as male 86 (74.8%) were reached; and the 1 patient who self-identified as non-binary was reached. The age of selected patients ranged from 19 to 83 years old, with a median age of 52 years old. Rate of successful outreach attempt did not differ by age-group with 76% of contacted patients successfully reached in each group. Sixty of the patients selected for calls (27.4%) had a preferred health care language other than English, including Spanish, Portuguese, or Haitian Creole. Of the non-English speaking patients who were successfully contacted, 95.6% were reached in their preferred health care language.

Themes identified by students were divided into the observed value for the three different stakeholders in the educational triad: patients, students, and providers (Table 1).

**Table 1:** Value for each stakeholder in educational triad

Patient	Student	Provider
Identification of social needs and connection to local services	Meaningful role on care team	Enhanced care for vulnerable patients
Personalized education and health promotion	Telehealth skills practice	
Engagement for socially isolated patients	Practical education addressing SDOH	
	Interprofessional learning community	
	Targeted content education (Covid-19 updates)	

### Value to patients

1. Identification of social needs and connection to local services. Our team connected patients facing food insecurity with food resources in their local community through a Boston-wide hotline in multiple languages. In addition, a clinic-based social worker spoke to all patients struggling with food or financial insecurity to determine if they qualified for food or rental assistance.
2. Personalized education and health promotion. Phone calls with patients included education around Covid-19 using a harm reduction framework. Patients had the opportunity to ask questions about Covid-19, raise concerns around the interaction of HIV and SARS-CoV-2, and obtain necessary information to ensure decreased transmission of Covid-19 (e.g. effective social distancing, safer grocery shopping).
3. Engagement for socially isolated individuals. In response to the guided question about physical and emotional safety of the home environment (Additional file 1, question 7), many patients identified loneliness as their primary challenge, and reported that the phone calls reinforced their connection to the clinic, and made them feel less socially isolated.

### Value to students

1. Meaningful role on care team Students consistently reported that their role on the care team felt authentic and valuable. They were able to identify needs that, without the program, would have gone unnoticed. As the primary touch point for patients, they were able to take a leading role in designing the project and altering the script to meet the evolving needs of patients.
2. Telehealth skills practice. The program provided an experiential opportunity to learn and practice telehealth skills including the formation of a therapeutic alliance without the benefit of sharing physical space or visual cues.
3. Practical education addressing SDOH. Students noted that this program provided an opportunity to apply social medicine concepts learned in pre-clerkship curricula into clinical practice. Students received structured training on how to create space for patients to engage in conversations about social factors that impact health, including specific language and communication skills to elicit patient experience with structural inequities.
4. Interprofessional learning community. Through the structured longitudinal collaboration with both a licensed social worker and a physician, medical students had the opportunity to engage in interdisciplinary care. The weekly team meeting provided space for peer support, education, and feedback on strategies to engage with patients around structural drivers of health. The students received feedback from the faculty leads and their peers on their documentation, communication skills and patient engagement strategies.
5. Covid-19 learning. Students had weekly interactive case-based didactics on Covid-19 facilitated by an infectious disease physician. The sessions kept students at the forefront of the emerging literature of this novel pathogen. Students had the opportunity to critically review the evolving understanding of the disease and discuss how to integrate new evidence into their care for patients.

### Value to providers

1. Enhanced care for vulnerable patients. The student outreach calls extended the clinic’s reach during a period when many patients were at risk for being lost to follow up. This program complemented the work of clinicians and social workers by reaching patients, ensuring their safety, and helping to connect them to food, housing, financial and educational resources.

## Discussion

Historically at risk for stigma and social isolation, patients with HIV are particularly vulnerable to the dual forces of economic hardship and physical distancing imposed by the Covid-19 pandemic [8]. Student outreach calls focused on SDOH created an opportunity for proactive engagement and identification of social needs outside the structure of a traditional office visit. We strove to achieve language concordance which may facilitate the model of reflective listening and partnership-building. A consistent theme that emerged during calls was the prevalence of loneliness and isolation. Although the impact of the calls on loneliness was not directly measured during our project, a recent study demonstrated that outreach calls by laypeople can decrease loneliness, depression, and anxiety in older adults [11]. The program created infrastructure to identify and address social, medical, and emotional needs of a vulnerable population.

In parallel, the program provided an opportunity for students to engage as important members of a care team during a time when they were displaced from traditional clinical clerkships. In the modern era of medical education, students are often viewed as observers and learners rather than value-added participants in the care team [12]. Leaders in education reform recognize that students can make meaningful, recognizable contributions during their undergraduate medical education [13]. Students played an integral role in designing the outreach program, were the essential members of the care team making calls, and guided changes to the process to meet the evolving needs of patients.

The program also created an opportunity for students to practice clinical skills including interacting with patients using virtual tools, and engaging patients in conversations around SDOH. Multiple studies have shown that SDOH curricula are most effective when they include experiential learning [14, 15], and as telehealth increasingly becomes a component of clinical care it is crucial for students to learn to effectively communicate with patients in this setting [16].

Finally, the program also gave the students the opportunity to work with an interprofessional team including a physician and social worker who were learning with them, as the Covid-19 landscape was ever-changing. This opportunity to co-learn and to see how professional teams manage uncertainty is invaluable.

### Limitations

There are several limitations to our study. First, we presented data from a single institution with a small cohort of students during a global pandemic which may limit generalizability of our data. However, we believe the same model can be integrated into different clinic settings and modified based on clinic structure and needs of patients. Second, the program relies on patients having access to a phone so may not be generalizable to clinics that care for under-resourced patients with limited access to this technology. Third, we did not perform a formal evaluation of specific outcomes for patients or students. Identification of themes relied on patient and student report which could be impacted by availability bias or social-desirability bias. Moreover, these students volunteered to participate in this program, so their experience may not be generalizable to all medical students. Evaluation of specific process and outcome measures would be important to guide future implementation.

### Next steps

The outreach program can be adapted to meet the shifting needs of patients and learners across different settings. For example, by modifying our script, we used the same student-led telehealth outreach model to engage patients in our HIV clinic in conversations around Covid-19 vaccine hesitancy with the goal of increasing vaccine access and uptake in our clinic population. This model can become sustainable by integrating it into longitudinal ambulatory experiences. Using our script as a template, the program can be adapted in other ambulatory settings, and students can play a leading role in population health management across different health conditions.

Finally, while we demonstrated the feasibility of a pilot outreach program, we plan to evaluate the program further to measure specific outcomes for both patients and learners.

## Conclusion

As clinicians, educators, and students of the twenty-first century, we have an opportunity to modernize medical education and prepare students for careers in medicine that will undoubtedly have elements of virtual care [17]. We should strive to use virtual tools to create models of care that promote health equity for patients and authentic clinical experiences for students [18, 19]. Our proactive outreach program provides hands-on telehealth experience, engagement with patients around SDOH, and interprofessional education and supervision.

## Supplementary Information

**Additional file 1.**

## Data Availability

The datasets generated and/or analysed during the current study are strictly demographic and are available from the corresponding author upon request.
