# Supplementary material for: A student-led interprofessional virtual outreach program for people with HIV during the Covid-19 pandemic: a pilot program at an academic medical center in Boston
Source: BMC Med Educ. 2022 Sep 2;22:657. doi: 10.1186/s12909-022-03716-w (PMC9438880; doi:10.1186/s12909-022-03716-w)
Supplement: Supplementary file 1 — Additional file 1. [file 12909_2022_3716_MOESM1_ESM.docx]

**Appendix 1: Semi-structured Script**

Hi, my name is  ______________. I’m a medical student calling from Dr.  _____’s office. They asked me to call you to see how you are doing. Is this an ok time to talk for a few minutes? [In case their family is not aware they are engaged in health care/HIV care] Would there be a better time for me to call back?

1. How are you doing today?
2. We have found there is a lot of information about coronavirus from different sources that can leave many people feeling confused or even scared. What is your understanding of coronavirus and what you can do to lower your chances of getting it?
3. We are finding that many people are encountering challenges that they have never faced before.  For example, some people are struggling with access to food for the very first time in their lives.  What challenges have you faced?
4. Do you have any concerns about your access to enough food? [This can lead to referrals to food stamps, or providing the number for the Food Source Hotline where people can find out where food is available in their neighborhoods.]
5. Some people qualify for rent assistance though everyone’s circumstances are different. If you are renting, are you behind on your rent?
6. Do you have any other financial concerns?
7. You are spending so much time in your home, is home safe and comfortable [physically, emotionally] for you?
8. Are there other things or people that you are particularly worried about right now?
9. Do you have access to your medications? Do you need refills?
10. Do you have access to a mask for when you leave the house?  Did you know that you can purchase a mask at your local pharmacy?  Do you think you would be able to purchase one? [If not possible] Would you like us to send you a mask?

Legend: Semi-structured script was updated iteratively based on weekly feedback from student callers, and evolving patient needs during different points in the pandemic.
